# Generative Artificial Intelligence Use and Learning Engagement Among Medical Undergraduates: Statistical Indirect and Configurational Associations Involving Basic Psychological Need Satisfaction

**DOI:** 10.3390/bs16091694

**Published:** 2026-09-20

**Authors:** Qian He, Guanglei Chang, Ning Su, Yingying Yang, Jun Ma

**Affiliations:** 1 College of Public Health, Chongqing Medical University, Chongqing 401331, China; 2 University-Town Hospital of Chongqing Medical University, Chongqing 401331, China; 3 Department of Special Medical Service Center, The First Affiliated Hospital of Chongqing Medical University, Chongqing 400016, China

**Keywords:** generative artificial intelligence, medical undergraduates, learning engagement, basic psychological need satisfaction, fuzzy-set qualitative comparative analysis

## Abstract

As generative artificial intelligence (GAI) becomes increasingly integrated into undergraduate medical education, its relationship with students’ learning engagement warrants further investigation. Guided by self-determination theory, this cross-sectional questionnaire study surveyed 498 medical undergraduates at a medical university in China and used structural equation modeling (SEM) and fuzzy-set qualitative comparative analysis (fsQCA) to examine average associations and configurational patterns among GAI use, basic psychological need satisfaction, and learning engagement. SEM results showed that GAI use was positively but weakly associated with basic psychological need satisfaction, whereas basic psychological need satisfaction was more strongly associated with learning engagement. The direct association between GAI use and learning engagement was not statistically significant, while the statistical indirect association through basic psychological need satisfaction was significant but small. The fsQCA results showed that high GAI use was not a necessary condition for high learning engagement. The configuration combining autonomy, competence, and relatedness need satisfaction had the highest coverage and showed relatively stable results across threshold adjustments, with competence need satisfaction as a core condition. These findings suggest that the educational relevance of GAI use may be better understood in relation to students’ psychological need conditions than to use frequency alone.

## 1. Introduction

Generative artificial intelligence(GAI) is rapidly entering higher education and is increasingly influencing how students access information, understand knowledge, complete learning tasks, and prepare for academic activities. Unlike traditional digital learning tools that are primarily used for information retrieval or resource presentation, generative AI can provide immediate explanations, generate content, clarify questions, and offer task-related feedback through natural-language interaction, thereby creating new forms of support for student learning (Kasneci et al., 2023; Lo, 2023). However, the integration of artificial intelligence into education involves more than the adoption of a new technological tool; it also engages social–psychological processes related to learner motivation, perceived competence, self-regulation, and social relationships. Accordingly, understanding the relationship between generative AI use and learning outcomes requires attention not only to whether or how frequently students use such tools but also to the psychological and social conditions under which their use occurs.

Learning engagement is an important indicator of students’ positive learning states and is commonly reflected in vigor, dedication, and absorption during the learning process (Schaufeli et al., 2002). According to self-determination theory, sustained student engagement depends not only on external learning resources but also on whether the basic psychological needs for autonomy, competence, and relatedness are satisfied (Ryan & Deci, 2017, 2020; Vansteenkiste et al., 2020). In learning contexts supported by generative AI, technological tools can provide students with information and task-related assistance, but whether such support is associated with greater learning engagement may depend on whether students experience autonomous choice, task competence, and social connectedness during learning. It is therefore necessary to further examine the relationships among generative AI use, basic psychological need satisfaction, and learning engagement from a social–psychological perspective.

Medical undergraduates provide a particularly demanding learning context in which to examine these relationships. Undergraduate medical education is characterized by a high density of knowledge, a lengthy training process, and substantial professional responsibility. Students are required to continuously acquire complex knowledge while progressively developing competencies in evidence appraisal, clinical reasoning, and professional judgment. During this process, their motivation and engagement may be influenced by both the educational environment and their psychological experiences (Orsini et al., 2016). Generative AI may support medical undergraduates by providing knowledge explanations, assisting with literature synthesis, and facilitating problem analysis, but medical learning places particularly high demands on informational accuracy, evidence reliability, and professional judgment (Gilson et al., 2023; Kung et al., 2023; Sallam, 2023). Recent evidence also indicates that medical students are increasingly using generative AI to learn and review medical content, summarize clinical information, and support other academic tasks, while expressing substantial concerns about inaccuracy and bias (Tran et al., 2025). Therefore, in medical learning contexts, the relationship between generative AI use and learning engagement should be understood in conjunction with students’ motivational states, competence experiences, and social connectedness.

Existing research has substantially expanded our understanding of students’ perceptions, acceptance, and potential uses of generative AI, but several issues warrant further investigation. First, the frequency or breadth of generative AI use may not adequately reflect the quality, specific purposes, or educational value of such use. Second, the relationship between generative AI use and sustained learning engagement may vary according to students’ psychological experiences rather than depend solely on the extent of use itself. Third, variable-oriented approaches are well suited to estimating average associations across a sample but are comparatively limited in explaining how different technology-use conditions and psychological need conditions may combine in multiple ways to be associated with the same outcome of high learning engagement. These issues are particularly relevant in undergraduate medical education because medical students using AI-generated information must not only manage complex learning tasks but also evaluate evidence and maintain professional judgment. Accordingly, guided primarily by self-determination theory, this study combines covariance-based structural equation modeling (CB-SEM) with fuzzy-set qualitative comparative analysis (fsQCA) to examine both average structural associations among the study variables and multiple configurations associated with high learning engagement.

## 2. Literature Review and Theoretical Framework

### 2.1. Generative Artificial Intelligence Use in Medical Learning Contexts

Generative artificial intelligence can produce knowledge explanations, answers to questions, text drafts, and task-related suggestions through natural-language interaction in response to user prompts. It has gradually become integrated into university students’ coursework, academic writing, research activities, and personal development contexts (Lo, 2023). Existing research indicates that university students generally recognize the potential value of GAI for explaining complex concepts, expanding ideas, improving the efficiency of information acquisition, and supporting task completion, while also expressing concerns regarding accuracy, privacy, academic integrity, and overreliance (Chan & Hu, 2023). Compared with conventional digital tools that primarily retrieve and present existing information, GAI can generate content with a certain degree of contextual relevance based on user input. However, the quality of its outputs remains dependent on factors such as task characteristics, prompting strategies, and model capabilities, making verification, comparison, and judgment by learners necessary (Kasneci et al., 2023).

In the present study, GAI use refers to students’ overall extent of use across a broad range of learning- and personal development-related contexts, rather than use within a particular course or a single task. This conceptualization is consistent with the measurement instrument used in the study and captures students’ overall level of GAI use in coursework, research activities, everyday knowledge acquisition and problem-solving, and preparation for further study and career development. It should be emphasized that a higher overall level of use does not necessarily indicate higher-quality use or directly represent better learning outcomes.

For medical undergraduates, GAI may support knowledge explanation, literature synthesis, problem analysis, and learning-plan development. However, medical education places particularly high demands on evidence reliability, clinical reasoning, and professional responsibility (Gilson et al., 2023; Kung et al., 2023; Sallam, 2023). Medical students generally recognize the potential educational value of AI while remaining concerned about risks related to reliability, privacy, ethics, and appropriate integration into medical training (Duan et al., 2025). The significance of GAI in medical learning therefore cannot be evaluated solely on the basis of tool availability or frequency of use but should also be understood in relation to students’ ways of using the technology, their psychological states, and the social learning environments in which such use occurs.

### 2.2. Basic Psychological Need Satisfaction and Learning Engagement

Learning engagement refers to a positive, fulfilling, and persistent learning-related state and is commonly characterized by three dimensions: vigor, dedication, and absorption (Schaufeli et al., 2002). Undergraduate medical education involves a lengthy training period, a substantial knowledge load, and progressively increasing task demands. Students are therefore required to sustain their participation in knowledge integration, problem analysis, and self-directed learning. Research in health professions education has also shown that students’ motivation and engagement are jointly influenced by the educational environment, instructional support, and individual psychological experiences (Orsini et al., 2016).

Self-determination theory proposes that autonomy, competence, and relatedness are three basic psychological needs that support positive development and autonomous motivation (Ryan & Deci, 2017). Autonomy need satisfaction refers to experiencing choice, volition, and psychological freedom in one’s actions. Competence need satisfaction refers to feeling capable of completing tasks effectively and managing challenges. Relatedness need satisfaction refers to experiencing care, acceptance, and interpersonal connection from significant others and the social environment (Niemiec & Ryan, 2009). When these needs are satisfied, students are more likely to develop relatively autonomous forms of learning motivation and to sustain active participation and continued engagement (Vansteenkiste et al., 2020).

In GAI-related learning activities, basic psychological need satisfaction may provide an important psychological basis for understanding the relationship between GAI use and learning engagement. Students may use GAI to select questions independently, adjust their learning pace, and obtain task-related feedback, and these activities may be associated with experiences of autonomy and competence (Kasneci et al., 2023; Lo, 2023). However, when students primarily use the technology to generate answers rapidly or substitute for independent thinking, their active control and competence development may be constrained (Chan & Hu, 2023; Farrokhnia et al., 2024). Relatedness need satisfaction should not be attributed simply to the immediate responses provided by artificial intelligence, because students’ sense of relatedness continues to arise primarily from authentic relationships with teachers, peers, and learning communities. Accordingly, the three psychological needs may not only be individually associated with learning engagement but may also combine with GAI use in different ways that are jointly associated with high learning engagement.

### 2.3. Theoretical Framework and Complementary Analytical Perspectives

Self-determination theory provides the primary theoretical foundation for explaining the relationships among GAI use, basic psychological need satisfaction, and learning engagement in the present study. The theory emphasizes that satisfaction of the needs for autonomy, competence, and relatedness provides an important psychological foundation for autonomous motivation, sustained participation, and positive learning states (Ryan & Deci, 2017). From this perspective, the relationship between GAI use and learning engagement may depend not only on the extent of technology use itself but also on whether students are able to maintain a sense of autonomous choice, develop experiences of task competence, and preserve meaningful connections with teachers, peers, and the wider learning community during the learning process. Similar levels of GAI use may be accompanied by different psychological experiences and may therefore be associated with different levels of learning engagement. At the conceptual level, this study treats GAI use as a technology-related learning condition, basic psychological need satisfaction as a key learner-related psychological state, and learning engagement as a positive learning outcome.

Methodologically, structural equation modeling (SEM) was used to examine the overall structural associations among GAI use, basic psychological need satisfaction, and learning engagement, as well as the statistical indirect association involving basic psychological need satisfaction. Such variable-oriented methods are well suited to estimating average relationships across a sample but provide comparatively limited information on how multiple conditions may combine in different ways to be associated with high learning engagement. By contrast, fsQCA adopts a set-theoretic perspective to examine the necessity and joint sufficiency relationships between multiple conditions and a specific outcome (Ragin, 2008; Schneider & Wagemann, 2012). It can identify multiple equifinal configurations associated with the same outcome and reveal complementary or substitutive relationships among conditions within specific combinations (Fiss, 2011; Greckhamer et al., 2018).

**Figure 1:**
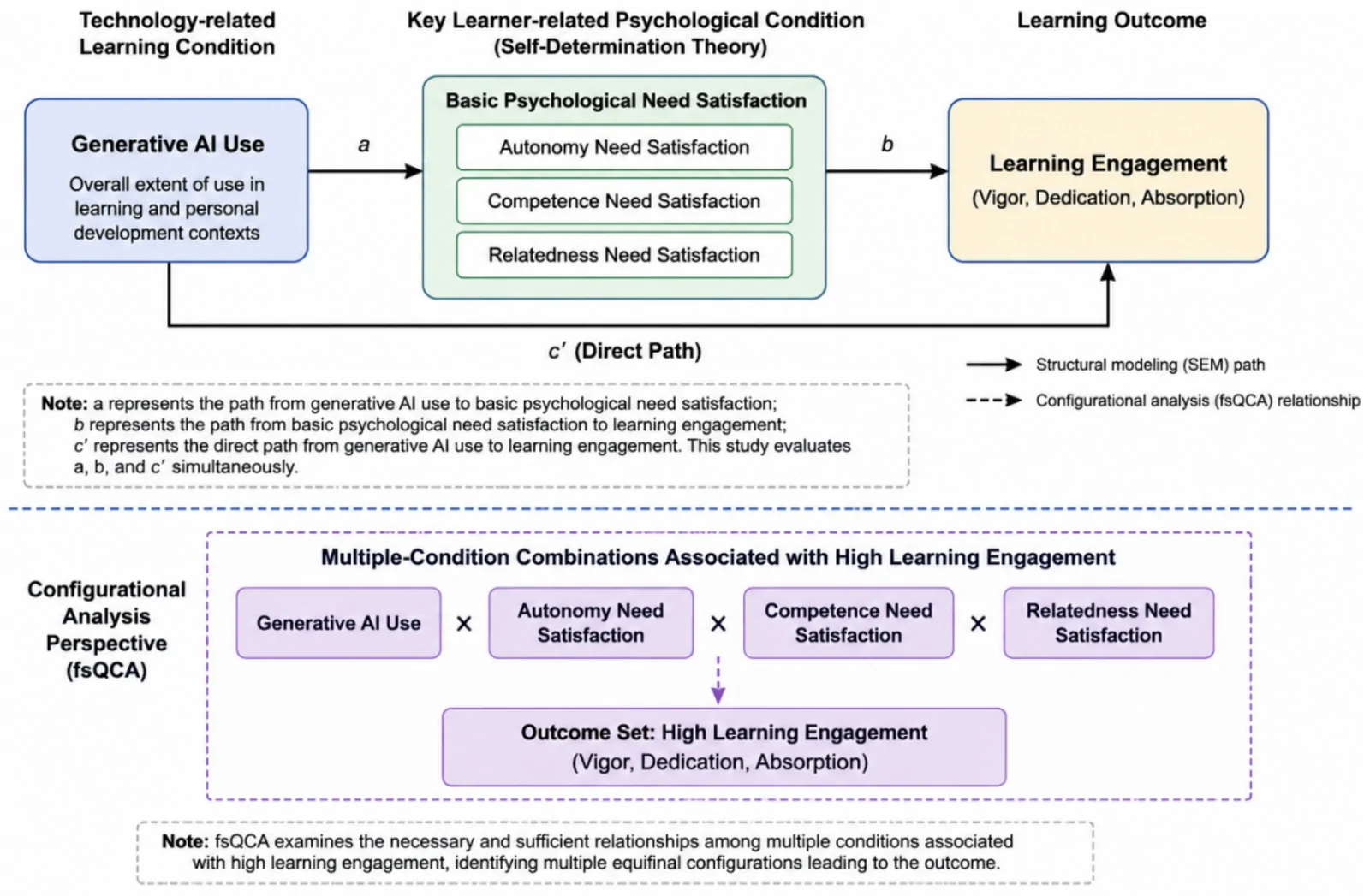
Theoretical framework of the relationships among generative artificial intelligence use, basic psychological need satisfaction, and learning engagement. **Abbreviations:** AI, artificial intelligence; SEM, structural equation modeling; fsQCA, fuzzy-set qualitative comparative analysis.

Accordingly, this study used SEM to examine the overall structural associations among the variables and fsQCA as a complementary approach to analyze multiple configurations associated with high learning engagement. This combined analytical strategy allowed the study to examine both average relationships across the sample and the associations between different technology–psychology condition combinations and high learning engagement. The theoretical framework is presented in Figure 1.

### 2.4. Research Questions

Based on the theoretical framework above, this study addressed the following research questions:

What are the associations of generative artificial intelligence (GAI) use with basic psychological need satisfaction and learning engagement among medical undergraduates?

Is GAI use statistically indirectly associated with learning engagement through basic psychological need satisfaction?

What configurations of GAI use and autonomy, competence, and relatedness need satisfaction are associated with high learning engagement?

## 3. Materials and Methods

### 3.1. Study Design

This study employed a cross-sectional questionnaire survey design and combined covariance-based structural equation modeling (CB-SEM) with fuzzy-set qualitative comparative analysis (fsQCA) to examine the relationships among generative artificial intelligence (GAI) use, basic psychological need satisfaction, and learning engagement among medical undergraduates.

CB-SEM was used as the primary variable-oriented analytical approach to estimate the overall structural associations among GAI use, basic psychological need satisfaction, and learning engagement, as well as the statistical indirect association involving basic psychological need satisfaction. As a complementary configurational approach, fsQCA was used to identify multiple combinations of GAI use, autonomy need satisfaction, competence need satisfaction, and relatedness need satisfaction associated with high learning engagement (Ragin, 2008; Schneider & Wagemann, 2012).

The two approaches were based on the same theoretical framework and set of variables but addressed different analytical questions. CB-SEM focused on average structural associations across the sample, whereas fsQCA examined set-theoretic relationships between different combinations of conditions and high learning engagement. Accordingly, this study adopted complementary quantitative analytical perspectives to examine both average variable-level associations and multiple configurational associations.

### 3.2. Participants and Data Sources

This study was conducted at a medical university in western China. The participants were undergraduate students enrolled in clinical medicine-related programs. Based on the classification of clinical medicine-related programs in the Catalogue of Undergraduate Programs in General Higher Education Institutions issued by the Ministry of Education of China, students from seven programs were included: Clinical Medicine, Anesthesiology, Medical Imaging, Ophthalmology and Optometry, Psychiatry, Radiology, and Pediatrics.

A convenience sampling method was used. Data were collected from December 2025 to January 2026. Participants were recruited primarily through classroom-based recruitment, with assistance from student affairs counselors, and The questionnaire was administered online using Sojump (Wenjuanxing, Changsha Ranxing Information Technology Co., Ltd., Changsha, China). Before participation, students were informed of the purpose of the study, the voluntary nature of participation, and the anonymous handling of their responses. A total of 569 eligible students were invited to participate, of whom 518 submitted questionnaires, yielding a response rate of 91.04%.

The inclusion criteria were as follows: (1) being enrolled as a full-time undergraduate student; (2) studying in one of the clinical medicine-related programs listed above; (3) being able to understand the questionnaire content; and (4) providing informed consent and voluntarily participating in the study. Questionnaires were excluded if they were not fully completed or displayed an invalid response pattern. An invalid response pattern was operationally defined as a run of identical responses extending across more than 20% of the questionnaire items.

Of the 518 submitted questionnaires, 20 were excluded during data-quality screening, leaving 498 questionnaires for the final analysis. This corresponded to a usable questionnaire rate of 96.14% among submitted questionnaires. All questionnaires retained in the final analytic sample were fully completed, and no item-level missing data remained.

The sample size was initially planned with reference to the commonly used heuristic of five to ten participants per questionnaire item. The full survey contained 67 items in total, including demographic and general GAI-use background questions as well as the items from the three primary measurement instruments, corresponding to a pragmatic target range of 335–670 participants. This heuristic was used as a practical reference for initial sample-size planning rather than as a model-specific power calculation for the SEM.

All participants voluntarily took part in the survey after providing informed consent. The questionnaires were completed anonymously, and all collected data were used solely for academic research purposes.

### 3.3. Measures

#### 3.3.1. Generative Artificial Intelligence Use

GAI use was measured using the “GAI Use in Typical Scenarios” section of the Questionnaire on Generative Artificial Intelligence Use among College Students (Y. Li et al., 2024). The original questionnaire comprises four parts: student demographic information, general background information concerning GAI use, GAI use in typical scenarios, and an open-ended question. In the present study, the 17-item “GAI Use in Typical Scenarios” section was adopted as the measure of GAI use and was the only section included in the primary GAI-use analysis. The original open-ended question was not included. The 17 items cover four typical scenarios: coursework, research activities, daily life, and further study and employment.

The 17-item GAI-use section was administered in Chinese, with no substantive wording modifications made for the present medical undergraduate sample. The present study used these items to assess students’ overall extent of GAI use across learning- and personal development-related contexts. Each item was rated on a five-point Likert scale, with total scores ranging from 17 to 85. Higher scores indicated a higher overall level of GAI use.

This measure reflects the overall extent of GAI use but does not directly assess specific use strategies, interaction quality, feedback quality, or use outcomes. In the SEM analysis, the total score across the 17 items was included as an observed independent variable. This approach was used because the instrument was developed primarily to describe GAI use across typical scenarios rather than to establish a validated multidimensional latent structure. By contrast, basic psychological need satisfaction and learning engagement were modeled as latent constructs using their respective dimension scores as indicators. In the fsQCA analysis, the item mean score was calculated and calibrated as a fuzzy-set condition. In the present study, Cronbach’s α for this section was 0.883.

#### 3.3.2. Basic Psychological Need Satisfaction

Basic psychological need satisfaction was assessed using the Chinese version of the Basic Psychological Need Satisfaction Scale. The scale was originally developed by Gagné and was subsequently adapted into Chinese to assess individuals’ general satisfaction of basic psychological needs (Gagné, 2003; Yu et al., 2012).

The scale contains 21 items across three dimensions: autonomy need satisfaction, competence need satisfaction, and relatedness need satisfaction. Each item is rated on a seven-point Likert scale. Items 3, 4, 7, 11, 15, 16, 18, 19, and 20 are reverse-scored. After reverse scoring, total scores range from 21 to 147, with higher scores indicating greater overall satisfaction of basic psychological needs.

It should be noted that the scale assesses participants’ general basic psychological need satisfaction rather than autonomy, competence, and relatedness experiences specifically arising from GAI use. In the present study, Cronbach’s α for the total scale was 0.923. Cronbach’s α values for autonomy need satisfaction, competence need satisfaction, and relatedness need satisfaction were 0.763, 0.781, and 0.833, respectively, indicating acceptable internal consistency for the total scale and its three dimensions.

In the SEM analysis, the dimension scores for autonomy need satisfaction, competence need satisfaction, and relatedness need satisfaction were used as indicators of the latent construct of basic psychological need satisfaction. In the fsQCA analysis, the item mean score for each dimension was calculated and used as an independent psychological condition.

#### 3.3.3. Learning Engagement

Learning engagement was assessed using the Chinese version of the Utrecht Work Engagement Scale–Student (UWES-S), revised by Fang et al. (2008). The scale was adapted by Schaufeli et al. from the original work engagement scale and includes three dimensions: vigor, dedication, and absorption, comprising 17 items in total (Schaufeli et al., 2002; Fang et al., 2008).

Each item is rated on a seven-point Likert scale, with total scores ranging from 17 to 119. Higher scores indicate greater learning engagement. In the present study, Cronbach’s α for the total scale was 0.951. Cronbach’s α values for vigor, dedication, and absorption were 0.900, 0.890, and 0.886, respectively, indicating good internal consistency for the total scale and its three dimensions.

In the SEM analysis, the dimension scores for vigor, dedication, and absorption were used as indicators of the latent construct of learning engagement. In the fsQCA analysis, the mean score across the 17 items was calculated and calibrated as the outcome set of high learning engagement.

### 3.4. Variable-Oriented Analysis Using SEM

First, descriptive statistics were calculated for the sample characteristics and major study variables. Categorical variables were summarized using frequencies and percentages, whereas continuous variables were described using means, standard deviations, minimum values, and maximum values. Because the distributions of some major variables deviated from normality, Spearman rank-order correlation analysis was used to examine the bivariate associations among GAI use, basic psychological need satisfaction, and learning engagement. Analyses were conducted using R version 4.4.2 (R Foundation for Statistical Computing, Vienna, Austria).

Subsequently, CB-SEM was conducted using the lavaan package in R (Rosseel, 2012). The total score for GAI use was included as an observed independent variable, whereas basic psychological need satisfaction and learning engagement were specified as latent variables. Basic psychological need satisfaction was measured using the dimension scores for autonomy need satisfaction, competence need satisfaction, and relatedness need satisfaction. Learning engagement was measured using the dimension scores for vigor, dedication, and absorption.

Before testing the structural paths, confirmatory factor analysis was performed to evaluate the two-factor measurement model comprising basic psychological need satisfaction and learning engagement. Standardized factor loadings and overall model fit indices were jointly examined to determine whether the measurement structure was consistent with the theoretical specification. Detailed standardized factor loadings and the latent correlation between basic psychological need satisfaction and learning engagement are presented in Supplementary Table S2.

The structural model specified the pathway from GAI use to basic psychological need satisfaction and subsequently to learning engagement. The model was used to examine the overall structural associations among the three variables and the statistical indirect association involving basic psychological need satisfaction. Because the distributions of some variable scores deviated from normality, robust maximum likelihood estimation (MLR) was used to estimate the measurement model, structural paths, robust standard errors, and robust model fit indices.

Gender, year of study, and academic program were included in the structural model as categorical control variables and were dummy-coded. Male students, first-year students, and students enrolled in Clinical Medicine were used as the respective reference groups. Because the number of fifth-year students was small, fourth- and fifth-year students were combined into a senior-year group to reduce the influence of sparse categories on parameter estimation.

Model fit was evaluated using the scaled chi-square statistic, robust comparative fit index (CFI), robust Tucker–Lewis index (TLI), robust root mean square error of approximation (RMSEA) with its 90% confidence interval, and standardized root mean square residual (SRMR) (Hu & Bentler, 1999). Because individual fit indices may be influenced by sample size, model degrees of freedom, and model complexity, model fit was evaluated comprehensively based on multiple indices.

The MLR estimates were treated as the primary structural model results. Because conventional nonparametric bootstrapping cannot be directly combined with MLR estimation in lavaan, the model was subsequently re-estimated using conventional maximum likelihood estimation while retaining the same measurement structure, structural paths, and control-variable specifications. A total of 5000 bootstrap resamples were then used to obtain percentile-based 95% confidence intervals for the direct association, statistical indirect association, and total association. An association was considered statistically significant when its 95% bootstrap confidence interval did not include zero (Preacher & Hayes, 2008).

The maximum likelihood bootstrap analysis was conducted primarily to provide a supplementary assessment of uncertainty in the association estimates, whereas interpretation of the main structural paths was based on the MLR model results.

Given the cross-sectional design of the present study, the statistical indirect association was used only to describe a pattern of relationships consistent with the theoretical model. It cannot establish temporal ordering or a causal mechanism among the variables (Maxwell & Cole, 2007).

### 3.5. Data Calibration for fsQCA

fsQCA requires raw variables to be transformed into fuzzy-set membership scores ranging from 0 to 1. In the present study, item mean scores were first calculated for each scale or dimension. Direct calibration was then applied by specifying three qualitative anchors: full non-membership, the crossover point, and full membership (Ragin, 2008; Schneider & Wagemann, 2012). The calibration anchors were primarily determined according to the semantic meaning of the response options and the theoretical midpoint of each scale, while the sample distributions were also examined to confirm that the anchors provided basic differentiation among cases. Inspection of the calibrated membership distributions further showed that cases were distributed across different levels of set membership without pronounced concentration around the crossover point, providing additional empirical support for the selected anchors.

GAI use was rated on a five-point Likert scale. Its anchors for full non-membership, the crossover point, and full membership were set at 2.0, 3.0, and 4.0, respectively. Autonomy need satisfaction, competence need satisfaction, relatedness need satisfaction, and learning engagement were rated on seven-point Likert scales, and their three calibration anchors were set at 2.5, 4.0, and 5.5, respectively. The calibration anchors are presented in Table 1.

A fuzzy-set membership score of 0.5 represents the point of maximum ambiguity regarding set membership and may interfere with the unambiguous classification of cases in truth-table analysis (Ragin, 2008; Duşa, 2019). Therefore, membership scores exactly equal to 0.500 after calibration were technically adjusted to 0.501. This adjustment was used solely to prevent cases from remaining at the point of maximum ambiguity and did not alter the rank ordering or substantive meaning of the cases.

### 3.6. Configurational Analysis Using fsQCA

As a complement to the variable-oriented SEM analysis, fsQCA was conducted using the QCA package in R (Duşa, 2019). First, necessity analyses were performed separately for GAI use, autonomy need satisfaction, competence need satisfaction, and relatedness need satisfaction, as well as for the absence of each condition, in relation to high learning engagement.

A condition with a necessity consistency score of 0.90 or higher was regarded as a potentially necessary condition. Its substantive relevance was then evaluated jointly using the relevance of necessity (RoN) and necessity coverage (Ragin, 2008; Schneider & Wagemann, 2012). A condition with consistency of at least 0.90 but a low RoN value was not directly interpreted as a substantively meaningful necessary condition.

A truth table was then constructed using GAI use, autonomy need satisfaction, competence need satisfaction, and relatedness need satisfaction as antecedent conditions and high learning engagement as the outcome set. Considering the sample size of 498 cases and the inclusion of four antecedent conditions, and following established methodological recommendations, the case-frequency threshold was set at 10, the consistency threshold at 0.80, and the proportional reduction in inconsistency (PRI) threshold at 0.70. Truth-table rows that met all three thresholds were coded as producing the high-learning-engagement outcome and were included in the subsequent Boolean minimization analysis.

Based on self-determination theory, the presence of autonomy need satisfaction, competence need satisfaction, and relatedness need satisfaction was specified as directionally favorable for high learning engagement (Ryan & Deci, 2017). Because previous research has identified the potential learning-support value of GAI (Chan & Hu, 2023; Kasneci et al., 2023; Lo, 2023), a higher level of GAI use was also specified as a condition that might be favorable for high learning engagement.

This directional expectation was used only in counterfactual analysis for generating the intermediate solution. It did not presuppose that GAI use itself constituted a necessary condition, a sufficient condition, or an independent linear predictor of high learning engagement.

Boolean minimization was used to generate the parsimonious and intermediate solutions. Core and peripheral conditions were distinguished according to whether a condition appeared in the same state across the two types of solutions. A condition appearing in the same state in both the parsimonious and intermediate solutions was identified as a core condition, whereas a condition appearing only in the intermediate solution was identified as a peripheral condition.

For each configuration, consistency, PRI, raw coverage, and unique coverage were reported. Overall solution consistency, PRI, and coverage were also reported. Blank cells in the results table indicated that the corresponding condition was unconstrained within that configuration, meaning that either its presence or its absence was compatible with the configuration. A blank cell did not indicate the absence of the condition.

To assess the sensitivity of the configurational results to the truth-table thresholds, robustness analyses were performed. With all other analytical settings held constant, the consistency threshold was increased from 0.80 to 0.85, and the case-frequency threshold was separately increased from 10 to 15. The configurational solutions for high learning engagement were then re-estimated. Robustness was evaluated according to whether the main configurations were retained, whether the core or peripheral status of the conditions changed, and whether overall solution consistency and coverage showed substantial changes.

## 4. Results

### 4.1. Sample Characteristics and Descriptive Statistics of the Key Variables

A total of 498 valid questionnaires were included in the final analysis. The sample comprised 316 female students (63.45%) and 182 male students (36.55%). First-year students constituted the largest group, with 192 participants (38.55%), followed by second-year students, with 172 participants (34.54%). Clinical Medicine was the most frequently represented academic program, accounting for 292 participants (58.63%), followed by Anesthesiology with 60 participants (12.05%) and Pediatrics with 54 participants (10.84%). The demographic characteristics of the sample are presented in Table 2.

The descriptive statistics for the key study variables showed that the mean total score for GAI use was 51.89 ± 9.53, the mean total score for basic psychological need satisfaction was 103.65 ± 17.79, and the mean total score for learning engagement was 77.87 ± 17.15. The descriptive statistics for the key variables are presented in Table 3.

### 4.2. Common Method Bias Assessment and Correlation Analysis

The Harman single-factor test showed that the first unrotated factor explained 26.08% of the total variance, indicating that no single factor dominated the variance across all measures. However, given the limited diagnostic capacity of the Harman single-factor test, this finding could not completely rule out the possibility of common method bias (Podsakoff et al., 2003).

The Spearman correlation analysis showed that GAI use was significantly but weakly positively correlated with basic psychological need satisfaction (ρ = 0.109, *p* = 0.015) and learning engagement (ρ = 0.092, *p* = 0.040). Basic psychological need satisfaction was moderately and positively correlated with learning engagement (ρ = 0.426, *p* < 0.001). The correlation results are presented in Table 4.

### 4.3. Measurement Model and Structural Model Results

GAI use was entered into the structural model as an observed variable. The confirmatory factor analysis primarily evaluated the two-factor measurement structure comprising basic psychological need satisfaction and learning engagement. The standardized factor loadings for the six dimension scores ranged from 0.850 to 0.955, and all were statistically significant. The latent correlation between basic psychological need satisfaction and learning engagement was 0.530 (*p* < 0.001). Detailed results are presented in Supplementary Table S2. The measurement model yielded a robust CFI of 0.985, a robust TLI of 0.972, and an SRMR of 0.027. The robust RMSEA was 0.095, with a 90% confidence interval of [0.065, 0.126]. Although the RMSEA was relatively high, the remaining fit indices indicated good model fit. Given that RMSEA can perform less favorably in models with small degrees of freedom, the measurement model was evaluated comprehensively using multiple fit indices rather than relying on RMSEA alone (Kenny et al., 2015).

The structural model was subsequently estimated using robust maximum likelihood estimation. The model showed an overall good fit: scaled χ^2^(52) = 109.803, *p* < 0.001, robust CFI = 0.976, robust TLI = 0.963, robust RMSEA = 0.047, 90% CI [0.035, 0.059], and SRMR = 0.017.

The structural path estimates are presented in Table 5. After controlling for gender, year of study, and academic program, GAI use was significantly but weakly positively associated with basic psychological need satisfaction (*B* = 0.017, β = 0.155, *p* = 0.002). Basic psychological need satisfaction was strongly and positively associated with learning engagement (*B* = 0.612, β = 0.521, *p* < 0.001). The direct association between GAI use and learning engagement was small and did not reach statistical significance (*B* = 0.004, β = 0.035, *p* = 0.404). Overall, GAI use showed a weak positive association with basic psychological need satisfaction, whereas the association between basic psychological need satisfaction and learning engagement was more pronounced.

### 4.4. Direct, Statistical Indirect, and Total Associations

Building on the structural path analysis, the model was re-estimated using conventional maximum likelihood estimation while retaining the same measurement structure, structural paths, and control-variable specifications. A total of 5000 bootstrap resamples were used to estimate the direct, statistical indirect, and total associations between GAI use and learning engagement. The results are presented in Table 6.

The direct association did not reach statistical significance (*B* = 0.004, β = 0.035, *p* = 0.416, 95% bootstrap CI [−0.006, 0.016]). The statistical indirect association through basic psychological need satisfaction was statistically significant but small in magnitude (*B* = 0.010, β = 0.081, *p* = 0.003, 95% bootstrap CI [0.004, 0.017]). The total association was also statistically significant and small in magnitude (*B* = 0.015, β = 0.116, *p* = 0.020, 95% bootstrap CI [0.003, 0.027]).

These results indicate a statistically significant but weak overall association between GAI use and learning engagement. After controlling for gender, year of study, and academic program, the direct association was not statistically significant, whereas the statistical indirect association through general basic psychological need satisfaction reached statistical significance. Given the cross-sectional design and the fact that basic psychological need satisfaction was not measured specifically in relation to GAI use, these findings do not demonstrate that GAI use increases learning engagement by satisfying students’ psychological needs, nor do they establish the temporal ordering of the variables.

### 4.5. Configurational Analysis Results

As a complement to the variable-oriented SEM analysis, fsQCA was further used to examine the necessary conditions and sufficient configurations associated with high learning engagement. The results of the necessity analysis are presented in Table 7. Relatedness need satisfaction yielded a necessity consistency of 0.919, exceeding the commonly used threshold of 0.90. However, its RoN value was only 0.498, indicating relatively limited relevance as a necessary condition. The consistency for autonomy need satisfaction was 0.897, which approached but did not reach the 0.90 threshold. The consistency values for competence need satisfaction and GAI use were 0.873 and 0.645, respectively, both below the necessity threshold. The consistency values for the absence of each condition ranged from 0.239 to 0.585, all below 0.90. Overall, no single condition was interpreted as a strictly necessary condition for high learning engagement.

Following the necessity analysis, a sufficiency analysis was conducted to identify configurations associated with high learning engagement. Boolean minimization produced the following parsimonious solution:

COM + AUT × GAI → ENG

The intermediate solution identified three configurations associated with high learning engagement among medical undergraduates:

AUT × COM × REL + AUT × REL × GAI + COM × REL × GAI → ENG

In these expressions, AUT denotes autonomy need satisfaction, COM denotes competence need satisfaction, REL denotes relatedness need satisfaction, GAI denotes generative artificial intelligence use, and ENG denotes high learning engagement. By comparing the parsimonious and intermediate solutions, conditions appearing in the same state in both solutions were identified as core conditions, whereas conditions appearing only in the intermediate solution were identified as peripheral conditions. The sufficient configurations are presented in Table 8.

Configuration C1, labeled the comprehensive psychological need satisfaction configuration, consisted of the core presence of competence need satisfaction and the peripheral presence of autonomy and relatedness need satisfaction. GAI use was unconstrained in this configuration. C1 had a consistency of 0.832, a PRI of 0.757, a raw coverage of 0.829, and a unique coverage of 0.250. Both its raw coverage and unique coverage were the highest among the three configurations, indicating that it was the principal configuration with the broadest empirical coverage.

Configuration C2, labeled the autonomy–relatedness–GAI use configuration, consisted of the core presence of autonomy need satisfaction and GAI use, together with the peripheral presence of relatedness need satisfaction. Competence need satisfaction was unconstrained. C2 had a consistency of 0.868, a PRI of 0.785, a raw coverage of 0.599, and a unique coverage of only 0.020, indicating substantial overlap with the other configurations. Accordingly, C2 was interpreted primarily as a supplementary configuration beyond C1.

Configuration C3, labeled the competence–relatedness–GAI use configuration, consisted of the core presence of competence need satisfaction and the peripheral presence of relatedness need satisfaction and GAI use. Autonomy need satisfaction was unconstrained. C3 had a consistency of 0.876, a PRI of 0.794, a raw coverage of 0.587, and a unique coverage of 0.009. Given its limited unique explanatory coverage and the fact that it was not retained when the case-frequency threshold was increased, C3 was treated as an exploratory configuration.

The overall solution consistency was 0.822, the overall PRI was 0.746, and the overall solution coverage was 0.858. All three configurations included at least two dimensions of basic psychological need satisfaction. Relatedness need satisfaction appeared as a peripheral condition in all three configurations, but this did not mean that it constituted an independently necessary condition. GAI use was unconstrained in C1, appeared as a core condition in C2, and appeared as a peripheral condition in C3, indicating that its configurational relationship with high learning engagement depended on the accompanying psychological need conditions.

To assess the sensitivity of the configurational results to the truth-table thresholds, the consistency threshold and case-frequency threshold were further adjusted. When the consistency threshold was increased from 0.80 to 0.85, all three configurations were retained, and the overall solution consistency and coverage remained unchanged. When the case-frequency threshold was increased from 10 to 15, C1 and C2 were retained, whereas C3 no longer appeared. The overall solution consistency increased slightly from 0.822 to 0.824, while the overall solution coverage decreased from 0.858 to 0.849. These findings indicate that C1 and C2 were relatively stable across threshold adjustments, whereas C3 was sensitive to the case-frequency threshold and was therefore interpreted only as exploratory. Detailed results are provided in Supplementary Table S1.

## 5. Discussion

### 5.1. Main Findings and Overall Interpretation

Guided primarily by self-determination theory, this study used SEM and fsQCA to examine the relationships among generative artificial intelligence (GAI) use, basic psychological need satisfaction, and learning engagement among medical undergraduates enrolled in clinical medicine-related programs. The SEM results indicated that the associations involving GAI use were modest, whereas basic psychological need satisfaction showed a stronger association with learning engagement. The fsQCA results further showed that high GAI use was not necessary for high learning engagement, while psychological need conditions, particularly competence need satisfaction, played a more consistent role across the identified configurations.

Overall, these findings indicate that the association between GAI use and learning engagement cannot be understood simply in terms of how much students use GAI. Students’ psychological need satisfaction appears to be more closely related to engagement than GAI use itself. This is consistent with self-determination theory, which regards basic psychological need satisfaction as an important psychological foundation for supporting autonomous motivation and sustained engagement (Ryan & Deci, 2020; Vansteenkiste et al., 2020).

The findings also reflect the limited and conditional role of GAI in undergraduate clinical medicine-related education. GAI can support a range of learning activities in medical and health professions education, including knowledge clarification, information organization, and self-directed learning (Kasneci et al., 2023; Lo, 2023; Pham et al., 2025). However, technological convenience does not necessarily translate into sustained engagement in medical learning. Medical undergraduates must also progressively develop competencies in evidence appraisal, case analysis, clinical reasoning, and professional judgment. Because the present study measured the overall extent of GAI use rather than specific purposes of use, prompting strategies, content verification, or use quality, the weak association observed between GAI use and learning engagement is understandable. The educational value of GAI should therefore not be judged solely by frequency of use but should also be considered in relation to the quality and purpose of use, including whether it supports active thinking, evidence verification, and professional reasoning. Consistent with this interpretation, recent evidence suggests that the educational benefits of GAI in medical education may vary across learning outcomes and instructional contexts rather than being uniformly superior to traditional approaches (J. Li et al., 2025).

### 5.2. Statistical Indirect Association Through Basic Psychological Need Satisfaction

The SEM results showed that GAI use was positively but weakly associated with basic psychological need satisfaction, whereas basic psychological need satisfaction was more strongly associated with learning engagement. The statistical indirect association between GAI use and learning engagement through basic psychological need satisfaction was significant but small, while the direct association was not statistically significant. This pattern suggests that the relationship between GAI use and learning engagement may be more closely associated with students’ psychological experiences during learning than with the extent of GAI use alone.

Self-determination theory provides a useful framework for interpreting this pattern. Satisfaction of autonomy, competence, and relatedness needs is closely associated with autonomous motivation and sustained engagement in learning (Ryan & Deci, 2020; Vansteenkiste et al., 2020). From this perspective, GAI may be more educationally relevant when its use is accompanied by experiences of choice, capability, and connection. For example, GAI-supported concept clarification, information organization, or timely feedback may be beneficial when these functions help students understand and manage learning tasks, but their availability alone does not necessarily correspond to greater learning engagement.

The small magnitude of the indirect association should also be considered when interpreting the findings. Because the study was cross-sectional, the temporal ordering among GAI use, basic psychological need satisfaction, and learning engagement cannot be determined. In addition, basic psychological need satisfaction was assessed as a general construct rather than specifically within GAI-supported learning. Accordingly, the observed indirect association is best interpreted as a statistical pattern consistent with the proposed theoretical model rather than as evidence of a causal mediation process.

### 5.3. Configurational Roles of Basic Psychological Needs and GAI Use

The fsQCA findings likewise indicated a more consistent configurational role for psychological need satisfaction than for GAI use in relation to high learning engagement. The consistency score for relatedness need satisfaction exceeded 0.90, and relatedness appeared as a peripheral condition in all three configurations associated with high learning engagement, suggesting a broadly supportive role. However, its RoN value was only 0.498, indicating limited substantive relevance as a necessary condition. It should therefore not be interpreted as a substantively meaningful necessary condition. A more appropriate interpretation is that relatedness need satisfaction may constitute a broadly supportive social–psychological context associated with high learning engagement but is insufficient on its own to define the outcome. Previous research has likewise shown that social connectedness, perceived support, and a sense of belonging are closely related to sustained student engagement (Kahu & Nelson, 2018; Niemiec & Ryan, 2009).

In undergraduate clinical medicine-related education, relatedness need satisfaction may be experienced through teacher–student and peer interactions in classroom learning, group discussions, skills training, case analysis, and clinical learning. Although GAI can provide immediate responses, it cannot replace authentic interpersonal support arising from teacher feedback, peer collaboration, and clinical supervision. The fact that relatedness appeared primarily as a peripheral condition also suggests that it may be more likely to operate in combination with autonomy need satisfaction, competence need satisfaction, or GAI use.

The comprehensive psychological need satisfaction configuration had the highest raw and unique coverage and remained stable across different threshold specifications, making it the principal configuration identified in this study. In this configuration, competence need satisfaction was a core condition, autonomy and relatedness need satisfaction were peripheral conditions, and GAI use was unconstrained. This finding is consistent with self-determination theory, which proposes that the joint satisfaction of autonomy, competence, and relatedness supports positive learning states (Ryan & Deci, 2017), while further highlighting the importance of competence experiences. For medical undergraduates, competence is reflected not only in completing coursework but also in understanding disease mechanisms, analyzing case information, evaluating the quality of evidence, and gradually acquiring clinical skills. When facing learning tasks that are extensive, highly specialized, and cumulative, the development of a sense of mastery may be closely associated with vigor, dedication, and absorption. Notably, competence need satisfaction occupied a core position in this principal configuration, whereas GAI use was unconstrained, pointing to a relatively more stable role of competence need satisfaction in configurations associated with high learning engagement.

GAI use did not constitute a necessary condition for high learning engagement and was not included in the principal configuration with the broadest empirical coverage. In the autonomy–relatedness–GAI use configuration, both autonomy need satisfaction and GAI use were core conditions. This suggests that, for some students, GAI use may be more likely to be associated with high learning engagement when it co-occurs with a stronger sense of autonomous choice. In learning activities involving course objectives, case analysis, or literature-based tasks, students’ ability to decide independently whether to use GAI, at which stage to use it, and how to verify generated content may be more educationally meaningful than merely increasing the frequency of use. However, because this configuration had low unique coverage, it is more appropriately interpreted as a supplementary configuration.

In the competence–relatedness–GAI use configuration, competence need satisfaction was a core condition, whereas relatedness need satisfaction and GAI use were peripheral conditions. This configuration remained primarily grounded in students’ sense of capability and task mastery, with GAI use functioning as an auxiliary condition. Because the configuration had low unique coverage and was no longer retained when the case-frequency threshold was increased, it should be interpreted cautiously as exploratory. Overall, GAI use was not a universal condition, and its configurational association with high learning engagement may vary depending on students’ psychological need profiles.

### 5.4. Theoretical and Practical Implications for Undergraduate Clinical Medicine-Related Education

The present findings extend research on GAI in undergraduate medical education by showing that the extent of GAI use, psychological need satisfaction, and learning engagement are not related in the same way. GAI use showed only a weak association with basic psychological need satisfaction, the statistical indirect association with learning engagement was small, and the configurational analysis placed greater emphasis on psychological need satisfaction, particularly competence need satisfaction. Existing research has largely focused on the functional capabilities of GAI, student acceptance, potential benefits, and risks associated with its use (Chan & Hu, 2023; Kasneci et al., 2023; Lo, 2023). The present findings suggest that learning engagement cannot be explained solely by the extent of technology use but should also be understood in relation to autonomy, competence, and relatedness need satisfaction, as well as the corresponding combinations of technological and psychological conditions. Accordingly, research on GAI in undergraduate medical education may benefit from shifting its focus from whether students use GAI to the medical learning contexts and psychological conditions under which its use is more likely to accompany positive learning states.

The distinction between core and peripheral conditions further indicates that different psychological needs do not occupy equivalent positions across configurations associated with high learning engagement. Competence experiences held a core position in the principal configuration, suggesting that technology integration may be more meaningful when it supports students’ understanding and mastery of complex medical learning tasks. Autonomy need satisfaction and GAI use jointly appeared as core conditions only in a specific configuration, indicating that the educational significance of GAI use may depend partly on whether students experience a sense of autonomous choice. Relatedness need satisfaction, by contrast, may be more appropriately understood as a supportive background condition across configurations.

In practice, medical schools may place less emphasis on the frequency of GAI use alone when integrating GAI into teaching. Recent studies of medical students have similarly reported substantial interest in AI-supported learning alongside concerns about information reliability and inappropriate academic use (Abdelhafiz et al., 2025; Duan et al., 2025). The present findings suggest that GAI-related learning activities may be more appropriately considered in relation to curriculum stage and the nature of professional learning tasks. Teachers may allow students some discretion over whether and at which stages to use GAI, while encouraging them to articulate their purposes for use and the basis on which generated content is verified. In activities such as literature reading, case discussion, and clinical reasoning, approaches such as staged prompting, concept comparison, and formative feedback could be explored, together with the use of textbooks, clinical guidelines, and original research articles to verify and interpret AI-generated content. More broadly, autonomy-supportive and appropriately structured learning environments may help support students’ experiences of autonomy and competence (Jang et al., 2010; Niemiec & Ryan, 2009), while teacher feedback, peer discussion, and clinical supervision may remain important for relatedness and professional development. Medical schools in comparable regional and resource contexts could adapt these approaches to their curricular needs, available resources, and instructional capacity, while establishing appropriate standards for content verification and responsible GAI use. These practices should be evaluated further in future educational intervention studies.

### 5.5. Limitations and Future Research

This study has several limitations. First, the cross-sectional design did not allow the temporal ordering or causal direction among GAI use, basic psychological need satisfaction, and learning engagement to be determined. The statistical indirect association only reflected a pattern of relationships consistent with the theoretical model, and reverse or bidirectional relationships remain possible (Maxwell & Cole, 2007).

Second, convenience sampling was used, and the sample was drawn from a single medical university in western China. In addition, the distributions of participants across years of study and academic programs were not fully balanced. The findings should therefore be generalized cautiously to other regions or different types of medical institutions.

Third, the major study variables were measured using self-report questionnaires administered at the same time point, which may have introduced recall bias, social desirability bias, and common method bias (Podsakoff et al., 2003). The measure of GAI use reflected the overall extent of use across broad learning and personal development contexts but did not distinguish among specific purposes of use, use strategies, interaction quality, or methods of verifying generated outputs.

Fourth, the present study assessed general basic psychological need satisfaction rather than context-specific experiences of autonomy, competence, and relatedness within GAI-supported learning. Accordingly, the statistical indirect association should not be interpreted as evidence that GAI use itself directly satisfied students’ basic psychological needs.

Finally, the fsQCA results may have been influenced by the calibration anchors, truth-table thresholds, and sample structure. Although the principal configurations showed a degree of stability in the sensitivity analyses, further validation is needed using different samples and alternative calibration schemes.

Future research could use longitudinal, experience-sampling, or experimental designs to examine the temporal ordering and dynamic relationships among the variables. Multi-center and cross-regional samples could be used to test the reproducibility of both the structural associations and the configurational patterns. Future studies could also combine usage logs, learning-platform data, course performance, and interview data to distinguish more clearly among different purposes and qualities of GAI use. In addition, context-specific measures of psychological need satisfaction in AI-supported learning environments could be developed or adopted to improve contextual interpretability and reproducibility.

## 6. Conclusions

This study used SEM and fsQCA to examine the relationships among generative artificial intelligence (GAI) use, basic psychological need satisfaction, and learning engagement among medical undergraduates enrolled in clinical medicine-related programs. The SEM results showed that the total association between GAI use and learning engagement was statistically significant but small in magnitude, whereas the direct association was not statistically significant. The statistical indirect association through general basic psychological need satisfaction was significant. The fsQCA results further indicated that a high level of GAI use was not a necessary condition for high learning engagement. The configuration jointly comprising autonomy, competence, and relatedness need satisfaction had the broadest empirical coverage, with competence need satisfaction serving as a core condition.

Overall, basic psychological need satisfaction, particularly competence experiences, showed a more stable association with learning engagement than the overall extent of GAI use. When integrating GAI into undergraduate clinical medicine-related education, attention should not be limited to frequency of use. Greater emphasis should be placed on whether its use supports the understanding of medical knowledge, evidence verification, clinical reasoning, and students’ needs for autonomy, competence, and relatedness. Given that this was a single-center cross-sectional study, the findings require further validation in multi-center samples.

## Figures and Tables

**Table 1:** Calibration Anchors for the Fuzzy-Set Qualitative Comparative Analysis.

Variable	Full Non-Membership	Crossover Point	Full Membership
Generative artificial intelligence use	2	3	4
Autonomy need satisfaction	2.5	4	5.5
Competence need satisfaction	2.5	4	5.5
Relatedness need satisfaction	2.5	4	5.5
Learning engagement	2.5	4	5.5

Note. GAI, generative artificial intelligence. All variables were calibrated using item mean scores. GAI use was measured using a five-point Likert scale, whereas autonomy need satisfaction, competence need satisfaction, relatedness need satisfaction, and learning engagement were measured using seven-point Likert scales.

**Table 2:** Descriptive Statistics of the Sample Characteristics (*n* = 498).

Variable	Category	*n*	Percentage (%)
Gender	Male	182	36.55
	Female	316	63.45
Year of study	First year	192	38.55
	Second year	172	34.54
	Third year	35	7.03
	Fourth year	95	19.08
	Fifth year	4	0.8
Academic program	Clinical Medicine	292	58.63
	Anesthesiology	60	12.05
	Medical Imaging	40	8.03
	Ophthalmology and Optometry	18	3.61
	Psychiatry	17	3.41
	Radiology	17	3.41
	Pediatrics	54	10.84

**Table 3:** Descriptive Statistics of the Key Study Variables (*n* = 498).

Variable	Mean ± SD	Minimum	Maximum
GAI use	51.89 ± 9.53	26	85
Basic psychological need satisfaction	103.65 ± 17.79	64	147
Learning engagement	77.87 ± 17.15	23	119

**Table 4:** Spearman Correlations among the Key Study Variables.

Variable	GAI Use	Basic Psychological Need Satisfaction	Learning Engagement
GAI use	1		
Basic psychological need satisfaction	0.109 *	1	
Learning engagement	0.092 *	0.426 ***	1

Note: * *p* < 0.05; *** *p* < 0.001.

**Table 5:** Structural Model Path Coefficients.

Path	*B*	SE	β	*p*
GAI use → Basic psychological need satisfaction	0.017	0.005	0.155	0.002
Basic psychological need satisfaction → Learning engagement	0.612	0.063	0.521	<0.001
GAI use → Learning engagement	0.004	0.005	0.035	0.404

Note: The model controlled for gender, year of study, and academic program. Gender, year of study, and academic program were dummy-coded, with male students, first-year students, and students enrolled in Clinical Medicine serving as the respective reference groups. Fourth- and fifth-year students were combined into a senior-year group. *B* denotes the unstandardized path coefficient, SE denotes the robust standard error obtained using MLR estimation, and β denotes the standardized path coefficient.

**Table 6:** Decomposition of the Associations between GAI Use and Learning Engagement.

Association Type	*B*	Bootstrap SE	β	*p*	95% Bootstrap CI
Direct association	0.004	0.006	0.035	0.416	[−0.006, 0.016]
Statistical indirect association	0.010	0.003	0.081	0.003	[0.004, 0.017]
Total association	0.015	0.006	0.116	0.020	[0.003, 0.027]

Note: *B* denotes the unstandardized association estimate, Bootstrap SE denotes the standard error obtained from 5000 bootstrap resamples, β denotes the standardized association estimate, and CI denotes the percentile-based bootstrap confidence interval.

**Table 7:** Necessity Analysis for High Learning Engagement.

Condition	Consistency	RoN	Coverage	Interpretation
Autonomy need satisfaction	0.897	0.618	0.797	Did not constitute a necessary condition
~Autonomy need satisfaction	0.301	0.942	0.813	Did not constitute a necessary condition
Competence need satisfaction	0.873	0.680	0.814	Did not constitute a necessary condition
~Competence need satisfaction	0.336	0.924	0.792	Did not constitute a necessary condition
Relatedness need satisfaction	0.919	0.498	0.760	Consistency reached the necessity threshold, but RoN was low
~Relatedness need satisfaction	0.239	0.962	0.832	Did not constitute a necessary condition
GAI use	0.645	0.845	0.830	Did not constitute a necessary condition
~GAI use	0.585	0.853	0.814	Did not constitute a necessary condition

Note: The symbol “~” denotes the absence of the corresponding condition. When necessity consistency reached 0.90 or higher, necessity was evaluated jointly with RoN and necessity coverage.

**Table 8:** Configurations Associated with High Learning Engagement among Medical Undergraduates.

Configuration	Autonomy	Competence	Relatedness	GAI Use	Consistency	PRI	Raw Coverage	Unique Coverage
C1 Comprehensive psychological need satisfaction configuration	▪	●	▪		0.832	0.757	0.829	0.250
C2 Autonomy–relatedness–GAI use configuration	●		▪	●	0.868	0.785	0.599	0.020
C3 Competence–relatedness–GAI use configuration		●	▪	▪	0.876	0.794	0.587	0.009
Overall solution					0.822	0.746	0.858	—

Note: Autonomy, Competence, and Relatedness refer to autonomy need satisfaction, competence need satisfaction, and relatedness need satisfaction, respectively. ● indicates the presence of a core condition, and ▪ indicates the presence of a peripheral condition. A blank cell indicates that the corresponding condition was unconstrained within that configuration and does not indicate the absence of the condition. Core and peripheral conditions were determined by comparing the parsimonious and intermediate solutions. Unique coverage applies only to individual configurations and is therefore not reported for the overall solution.

## Data Availability

The data that support the findings of this study are available from the corresponding author upon reasonable request, subject to ethical and privacy restrictions.

## Supplementary Materials

The following supporting information can be downloaded at: https://www.mdpi.com/article/10.3390/bs16091694/s1, Table S1: Robustness Checks of the fsQCA Results. Table S2: Standardized factor loadings and latent correlation for the measurement model.
