# Supplementary material for: Generative Artificial Intelligence Use and Learning Engagement Among Medical Undergraduates: Statistical Indirect and Configurational Associations Involving Basic Psychological Need Satisfaction
Source: Behav Sci (Basel). 2026 Sep 20;16(9):1694. doi: 10.3390/bs16091694 (PMC13603777; doi:10.3390/bs16091694)
Supplement: Supplementary file 1 [file behavsci-16-01694-s001.zip › behavsci-4565591-supplementary.pdf]

Supplementary Table S1. Robustness Checks of the fsQCA Results

Robustness Check	Threshold Specification	C1 Comprehensive Psychological Need Satisfaction Configuration	C2 Autonomy–Relatedness–GAI Use Configuration	C3 Competence–Relatedness–GAI Use Configuration	Overall Consistency	Overall Coverage
Main analysis	n.cut = 10; incl.cut = 0.80; PRI = 0.70	Retained	Retained	Retained	0.822	0.858
Increased consistency threshold	n.cut = 10; incl.cut = 0.85; PRI = 0.70	Retained	Retained	Retained	0.822	0.858
Increased frequency threshold	n.cut = 15; incl.cut = 0.80; PRI = 0.70	Retained	Retained	Not retained	0.824	0.849

Note: n.cut denotes the case-frequency threshold; incl.cut denotes the consistency threshold; PRI denotes proportional reduction in inconsistency. “Retained” indicates that the corresponding configuration was reproduced under the alternative threshold specification.

Supplementary Table S2. Standardized Factor Loadings and Latent Correlation for the Measurement Model

Latent variable	Indicator / latent relationship	Standardized loading / correlation	p value
Basic psychological need satisfaction	Autonomy need satisfaction	0.955	<0.001
Basic psychological need satisfaction	Competence need satisfaction	0.889	<0.001
Basic psychological need satisfaction	Relatedness need satisfaction	0.857	<0.001
Learning engagement	Vigor	0.882	<0.001
Learning engagement	Dedication	0.895	<0.001
Learning engagement	Absorption	0.850	<0.001
Latent correlation	Basic psychological need satisfaction ↔ Learning engagement	0.530	<0.001

Note：Standardized factor loadings and the latent correlation were obtained from the confirmatory factor analysis. The latent correlation refers to the correlation between basic psychological need satisfaction and learning engagement.
